# IgE, IgG1, and IgG4 Reactivity to* Dermatophagoides pteronyssinus* Glycosylated Extract in Allergic Patients

**DOI:** 10.1155/2019/9840890

**Published:** 2019-07-29

**Authors:** Rafael de Oliveira Resende, Leandro Hideki Ynoue, Juliana Silva Miranda, Karine Cristine de Almeida, Deise Aparecida de Oliveira Silva, Monica Camargo Sopelete, Ronaldo Alves, Margareth Leitão Gennari-Cardoso, Ernesto Akio Taketomi

**Affiliations:** ^1^Laboratory on Thymus Research, Oswaldo Cruz Institute, Fiocruz, Rio de Janeiro, Brazil; ^2^Laboratory of Allergy and Clinical Immunology, Biomedical Science Institute, Federal University of Uberlândia, Uberlândia, Brazil; ^3^Department of Biological Sciences, State University of Santa Cruz, Ilhéus, Brazil

## Abstract

**Background:**

House dust mites are important allergen sources and some of these allergenic proteins may contain carbohydrate moieties, which are able to be isolated using lectins, as Concanavalin A (ConA). This study aimed to investigate allergenicity (IgE) and antigenicity (IgG1 and IgG4) of ConA-unbound and ConA-bound* Dermatophagoides pteronyssinus *(Dpt) crude extracts using sera of mite-allergic patients as well as inhibition capacity of antibody binding.

**Material and Methods:**

We obtained mannose-enriched and mannose-depleted fractions from Dpt by ConA affinity chromatography. Both ConA-bound and ConA-unbound fractions were evaluated by ELISA and Western Blotting for specific IgE, IgG1, and IgG4 reactivity with sera obtained from 95 mite-allergic patients (DP+) and 92 nonallergic (NA) subjects. Inhibition ELISA was used to assess cross-reactivity between Dpt extract and its fractions.

**Results:**

Among the DP+ patients, no difference was found between ConA-unbound and ConA-bound fractions regarding the levels of specific IgE, IgG1, and IgG4. Nonallergic subjects had the same levels of specific IgG1 to both ConA-unbound and ConA-bound fractions, although for specific IgG4, values were higher for ConA-bound. A positive correlation was found among specific IgE, IgG1, and IgG4 levels when Dpt was compared to ConA-unbound and ConA-bound fractions. Recognition of crude Dpt by IgE, IgG1, and IgG4 was highly inhibited by ConA-unbound and ConA-bound fractions. Western Blotting revealed a broad spectrum of bands ranging from 14 to 116 kDa recognized by specific IgE and IgG4. However, IgG1 reached higher frequency values on high molecular weight polypeptides.

**Conclusion:**

ConA-unbound and ConA-bound fractions derived from* D. pteronyssinus *crude extract revealed important components involved in the IgE recognition in allergic patients as well as IgG1 and/or IgG4 in allergic and healthy subjects.

## 1. Introduction

House dust mites (HDM) have been described as important allergen sources causing respiratory allergies, such as asthma and rhinitis, and this association has led to the realization of several studies aiming to elucidate the role of the exposure to these allergens on the synthesis of components of the immune system, including cytokines, such as IL-4, IL-5, and IL-13 as well as different immunoglobulin classes [1]. In prevalence studies, mites from Pyroglyphidae (particularly* Dermatophagoides pteronyssinus* and* Dermatophagoides farinae*) and Echimyopodidae (*Blomia tropicalis*) families have been found in several countries, including areas in Latin America [2–5]. Allergens derived from these mites are characterized according to their biochemical and structural properties in association with the mechanism of IgE induction in the allergic response [6, 7]. More than 30 allergens from HDM have been described so far and Der p 1 and Der p 2, from* D. pteronyssinus*, are responsible for more than 50% of IgE reactivity in patients sensitized to HDM [8], although these major allergens present distinct biochemical features. Der p 1 is a glycosylated protein with cysteine-protease function that exhibits high identity to Der f 1, from* D. farinae*, and it is involved in the barrier disruption and Th2 cell differentiation [9] while Der p 2 is likely associated with Toll-like receptors, particularly TLR-4, by triggering components in the innate immunity [10]. Consequently, the association of these findings and the use of these proteins on diagnosis and treatment of the allergic diseases have determined most of the approaches in clinical immunology.

The contribution of IgG antibodies on the allergic response has also been investigated, although the mechanism of activation as well as interaction with allergens is controversial. Correlations between specific IgG and IgE antibodies propose that IgG is similarly important in the immune response triggered by airborne allergens, suggesting that IgE should not be considered an exclusive marker for the allergy status [11]. In addition to that, IgG4 is an important marker for immunotherapy efficacy and may act as a blocking element in the allergen presentation while IgG1 should be involved in the upregulation of the IgE/B cell binding complex [12]. Despite this, reports using IgG and IgG1/IgG4 subclasses as an alternative or complementary tool for allergy diagnosis either determination of predictive values have not been consistent [13].

Some allergenic extracts may contain carbohydrate molecules associated with proteins with a wide-ranging immune response profile, including IgE recognition [14–16]. The isolation and purification of these glycoconjugates have been frequently conducted using plant lectins as a ligand, which includes Concanavalin A (ConA) [17–19], and IgE reactivity to these components has been investigated in some allergen sources [20, 21]. Nevertheless, only few studies have been conducted aiming to investigate IgE reactivity to glycosylated components derived from HDM. The first* D. farinae* glycoconjugates were isolated by ConA-Sepharose affinity chromatography [22], and later, this technique was also used to isolate a high molecular weight antigen from* D. pteronyssinus *[23]. However, none of these studies demonstrated an IgE/IgG-binding profile against HDM crude extract as well as ConA-related fractions neither inhibition assays. Thus, we sought to investigate allergenicity (IgE) and antigenicity (IgG1 and IgG4) of ConA-bound and ConA-unbound* D. pteronyssinus *crude extracts using sera of mite-allergic patients and healthy subjects as well as inhibition capacity of antibody binding.

## 2. Materials and Methods

### 2.1. Crude Mite Extracts

*D. pteronyssinus* (Dpt),* D. farinae* (Df), and* B. tropicalis* (Bt) crude extracts used for serologic tests were prepared as previously described [24]. In brief, mite bodies were diluted in 5 mM borate-buffered saline (BBS, pH 8.0) containing protease inhibitors (50 *μ*g/ml leupeptin, 1 mM phenylmethylsulfonyl fluoride [PMSF], 1 mM benzamidine, and 10 *μ*g/ml aprotinin, Sigma Chemical Co., St. Louis, USA) and then exhaustively triturated in presence of liquid nitrogen. After 18h extraction at 4°C under orbital agitation, the extract was centrifuged at 30,000 × g, then supernatant was collected and dialyzed in 0.01 M phosphate-buffered saline (PBS, pH 7.2) in Amicon system (W.R. Grace & Co., Beverly, USA) with 10 kDa cut-off membrane. Protein and carbohydrate contents were determined by the Lowry [25] and Dubois [26] methods, respectively.

### 2.2. Concanavalin A-Sepharose Affinity Chromatography

ConA-Sepharose 4B matrix (5 ml; GE Healthcare, Uppsala, Sweden) was packed and equilibrated according to manufacturer instructions. To prevent ConA subunit coelution, matrix was treated with 25% glutaraldehyde as described elsewhere [27]. Dpt extract (5 mg protein) was loaded onto the column and washed with binding buffer containing 1 mM MgCl_2_, 1 mM MnCl_2_, and 1 mM CaCl_2_ in PBS while absorbance was monitored by UV at 280 nm. Each 2 ml-fraction was collected at 4°C and pooled, labeling ConA-unbound fraction. Once the fractions have reached background levels, 0.1 M methyl *α*-D-mannopyranoside (Ferro Pfanstiehl Laboratories, Inc., Waukegan, USA) was added and ConA-bound fractions were eluted and pooled. Protein and carbohydrate contents were also determined in the pooled fractions.

### 2.3. Measurement of Major Allergens

Der p 1 and Der p 2, Der f 1, and Blo t 5 major allergens content was measured in Dpt and ConA-unbound/bound fractions. High affinity microplates were coated with monoclonal antibodies (Indoor Biotechnologies, Charlottesville, USA), anti-Der p 1 (clone 5H8), anti-Der p 2 (clone 1D8), anti-Der f 1 (clone 6A8), and anti-Blo t 5 (clone 4G9) for 18h at 4°C, and blocked with PBS containing 0.05% Tween 20 and 1% bovine serum albumin (PBS-T-BSA). Dpt extract and reference standards (Der p 1-UVA93/02 and Der p 2-UVA92/01, University of Virginia, USA; STDF1, Indoor Biotechnologies; S-BT5, Indoor Biotechnologies) were added in 2-fold dilutions and biotinylated antibodies (Indoor Biotechnologies), anti-group 1 (clone 4C1), anti-group 2 (clone 7A1), and anti-Blo t 5 (clone 4D9), were used to detect the allergens. After incubation streptavidin-peroxidase (Sigma) assay was developed by ABTS containing 0.03% H_2_O_2_ and the reaction was read at 405 nm.

### 2.4. Subjects and Skin Prick Test (SPT)

A total of 187 subjects were recruited at the Clinics Hospital, Federal University of Uberlândia, Brazil, and submitted to SPT with Dpt, Df, and Bt extracts at 2 mg/ml in PBS added with 0.4% phenol and 50% glycerol (FDA Allergenic, Rio de Janeiro, Brazil). Histamine (10 mg/ml) and buffered glycerine-saline solution were used as positive and negative controls, respectively (FDA Allergenic). Wheal sizes 3 mm larger than the negative control were considered positive. Blood samples were obtained from all subjects and sera were stored at –20°C until serologic evaluations.

Patients with respiratory allergy (n=95; 35 men and 60 women; age: 25 ± 16 years) were grouped based on their history of rhinitis and asthma, physical examination, and SPT positivity to* D. pteronyssinus *(DP+ group). Healthy subjects (n=92; 22 men and 70 women; age: 28 ± 22 years) were included in the nonallergic (NA) group, based on the negative SPT results to airborne allergens and absence of clinical symptoms. None of the participants underwent previous allergenic immunotherapy. The study was conducted under approval of the local ethics committee and informed consent was obtained from all participants.

### 2.5. D. pteronyssinus-Specific IgE, IgG1, and IgG4 Levels

Specific IgE, IgG1, and IgG4 to Dpt as well as ConA-unbound and ConA-bound fractions were assessed by ELISA as previously described [24]. Briefly, microtiter plates were coated (1 *μ*g/well) with Dpt, ConA-unbound, or ConA-bound fractions, blocked with PBS-T-BSA, and incubated with serum samples diluted 1:2 in triplicate. Biotinylated anti-human IgE (1:500, Kirkegaard and Perry Laboratories Inc., Gaithersburg, USA), anti-human IgG1 (1:3000, Sigma), and anti-human IgG4 (1:3000, Sigma) were added followed by streptavidin-peroxidase (1:500, Sigma). ABTS and 0.03% H_2_O_2_ were used for color development and optical density was read at 405 nm. Results were expressed as ELISA index (EI), determined as follows: EI = absorbance of test sample/cut-off, where cut-off was calculated as the mean absorbance of negative control sera plus three standard deviations. Results for IgE, IgG1, and IgG4 were analyzed independently and EI above 1.2 were considered positive to exclude values close to EI = 1.0.

### 2.6. D. pteronyssinus-Specific IgE, IgG1, and IgG4 Inhibition ELISA

To evaluate cross-reactivity among Dpt extract and ConA-unbound and ConA-bound Dpt fractions as well as crude Df and Bt extracts, inhibition ELISA was conducted as previously described [24]. Dpt extract was loaded on the solid phase (1 *μ*g/well) and six pooled sera (diluted 1:5) selected from patients of the group DP+ were preadsorbed 18 h at 4°C with 10-fold-diluted Dpt extract and ConA-unbound and ConA-bound fractions as well as crude Df and Bt extracts as inhibitors. Tetanus toxoid (TT) was used as unrelated antigen. Residual uninhibited antibody reactivity to Dpt extract was measured by ELISA as described above and results were defined as percentage of reactivity to the respective extracts in the absence of inhibitors (inhibition %).

### 2.7. SDS-PAGE and Western Blotting

Polypeptides in Dpt extract and ConA-unbound and ConA-bound fractions were separated on sodium dodecyl sulphate-polyacrylamide gel electrophoresis (SDS-PAGE) under denaturing conditions and electrophoretic profile was visualized by silver staining. To identify IgE-, IgG1-, and IgG4-reactive polypeptide fractions in Dpt extract and ConA-unbound and ConA-bound fractions, 10 sera from the group DP+ and 10 sera from the group NA were selected for Western Blotting and performed as described elsewhere [24]. Briefly, Dpt extract and its fractions previously separated on 12% SDS-PAGE were electrotransferred onto a nitrocellulose membrane (Bio-Rad Laboratories, Inc., Hercules, USA) followed by blocking with 5% skimmed milk (SM) in 0.05% Tween 20 (PBS-T). Membrane was washed with PBS-T and incubated with serum samples diluted at 1:2 (for IgE and IgG1) or 1:5 (for IgG4) in PBS-T containing 1% SM for 18 h at room temperature. After washing with PBS-T, biotinylated anti-human IgE (1:250), anti-human IgG1 (1:1000), or anti-human IgG4 (1:100) antibodies were added and incubated for 2 h at room temperature followed by horseradish peroxidase-conjugated ABC Complex (DAKO Corp., Carpinteria, USA) diluted 1:250 for 1 h at room temperature. Reaction was revealed by adding tetrahydrochloride 3,3'-diaminobenzidine (DAB, Sigma).

### 2.8. Statistical Analysis

Differences between percentages were analyzed by the Chi-square test or Fisher's exact probability test, when appropriated. Differences in wheal sizes as well as specific IgE, IgG1, and IgG4 levels were assessed by the Kruskal-Wallis test followed by the Dunn's multiple comparison test. Correlations among levels of antibodies to Dpt extract and ConA-unbound and ConA-bound fractions were determined by the Spearman correlation test. Values of p < 0.05 were considered statistically significant.

## 3. Results

### 3.1. Analytical Profile of Dpt, ConA-Unbound, and ConA-Bound Fractions

**Figure 1:**
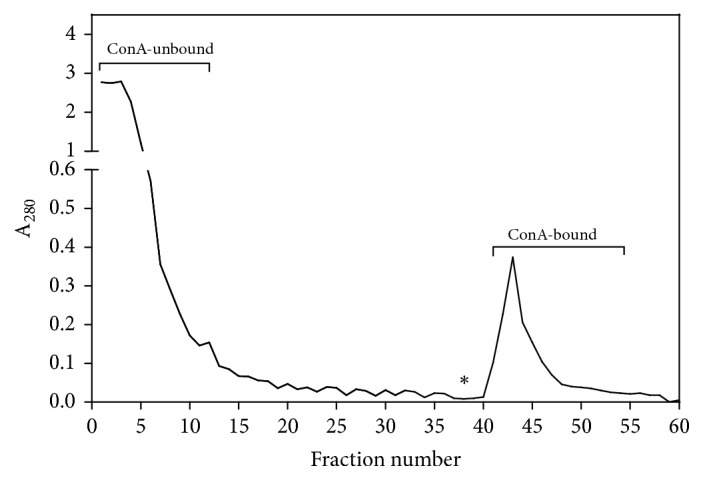
Fractionation profile of* D. pteronyssinus* crude extract (Dpt, 5 mg of total protein) on a ConA-Sepharose affinity column. ConA-unbound and ConA-bound fractions were eluted with binding buffer and 0.1 M methyl *α*-D-mannopyranoside (*∗*), respectively.

Fractionation of the Dpt extract (5 mg of total protein) on a ConA-Sepharose affinity column is demonstrated in Figure 1. Fractions 1 to 12 were pooled and labeled ConA-unbound fraction, which was obtained by elution with binding buffer. After a background level was reached (fractions 23 to 38), ConA-bound fraction (fractions 41 to 55) was eluted with 0.1 M methyl *α*-D-mannopyranoside. Total protein, carbohydrate, Der p 1, and Der p 2 contents in Dpt extract and ConA-unbound and ConA-bound fractions are shown in Table 1. ConA-bound fraction presented the lowest amount of total protein and carbohydrate (500 *μ*g/ml and 200 *μ*g/ml, respectively) and the highest protein/carbohydrate ratio (2.5), indicating that carbohydrate levels in relation to protein levels are lower in this fraction, comparing to ConA-unbound either Dpt extract. Although the difference between relative amounts of Der p 2 (6.1%) and Der p 1 (5.9%) in the Dpt extract was subtle, both ConA-unbound and ConA-bound fractions show higher levels of Der p 1 (10.3 % and 5.0 %, respectively) than Der p 2 (6.7% and 0.8%, respectively). As expected, only negligible levels of Der f 1 and Blo t 5 were found.

### 3.2. SPT

All allergic patients from the group DP+ had positive SPT results to* D. pteronyssinus *extract, although all of them were cosensitized to* D. farinae*. However, only 74.7% showed reactivity to* B. tropicalis* (p < 0.001). Wheal sizes were higher for* D. pteronyssinus *(median, interquartile ranges: 9.0, 7.5-10.5) than* D. farinae *(8.0; 6.0-10.5; p < 0.01), and* B. tropicalis *(5.0; 2.0-7.5; p < 0.001). Furthermore, wheal sizes for* B. tropicalis* were also lower when compared to* D. farinae* (p < 0.001).

### 3.3. IgE, IgG1, and IgG4 Antibodies to Dpt Extract and ConA-Unbound and ConA-Bound Fractions

**Figure 2:**
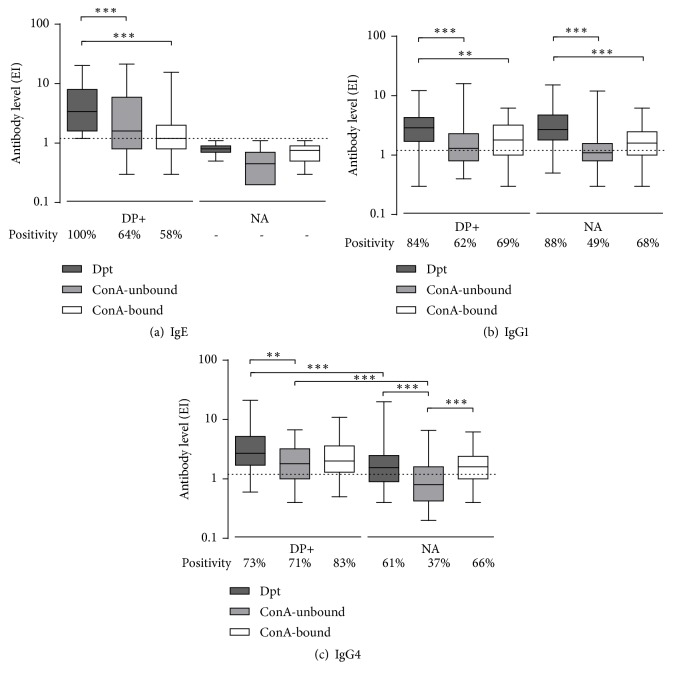
Levels of specific IgE (a), IgG1 (b), and IgG4 (c) to the* D. pteronyssinus *(Dpt) crude extract and ConA-unbound and ConA-bound fractions, expressed in ELISA index (EI) in sera from 95 patients with positive SPT to* D. pteronyssinus* (DP+ group) and 92 nonallergic (NA group) subjects. Boxes indicate the median with minimum and maximum values. The dashed line represents the positivity threshold value (EI = 1.2). Relative numbers of sera with EI above the positivity threshold value are also indicated (%). Significant differences in the EI are reported in indicative bars. *∗∗*p < 0.05; *∗∗∗*p < 0.001.

Specific IgE, IgG1, and IgG4 levels to Dpt, ConA-unbound, and ConA-bound fractions were determined in sera from both allergic patients (DP+ group) and nonallergic subjects (NA group) by ELISA (Figure 2). Specific IgE levels to Dpt extract and its fractions were significantly higher in sera from the group DP+ when compared to the group NA (p < 0.001). Allergic patients were included in the group DP+ based on their IgE positivity to Dpt extract; thus, all of 95 sera within this group reached specific IgE levels to the Dpt extract above the positivity threshold value (EI = 1.2) while only 61 and 40 serum samples had positive IgE to ConA-unbound and ConA-bound fractions, respectively (p < 0.0001). Noticeably, no serum sample had positive IgE within the group NA. In the group DP+, mean IgE levels to Dpt extract were higher than those to ConA-unbound or ConA-bound fractions (p < 0.001), although no significant difference was found between both fractions (Figure 2(a)).

Patients from the group DP+ had higher IgG1 levels to Dpt extract, when compared to ConA-unbound and ConA-bound fractions, with no significant difference between the fractions. A similar profile of these differences was observed in the group NA, although with a distinct statistical pattern (Figure 2(b)). Likewise, number of sera with positive IgG1 levels was higher in the Dpt comparing to both ConA-bound (p < 0.05) and ConA-unbound (p < 0.001) fractions in the group DP+ as well as in the group NA. When comparing both fractions, no difference was found between them in the group DP+. However, in the group NA, number of sera positive to ConA-bound fraction was higher than to ConA-unbound fraction (63* vs.* 45, respectively; p < 0.05).

In the group DP+, IgG4 mean levels to Dpt extract were higher than to ConA-unbound fraction (p < 0.01), but not to ConA-bound fraction. IgG4 positivity to ConA-unbound fraction (67 positive sera) was also lower than to ConA-bound fraction (79 positive sera) (p < 0.05). In the NA group, IgG4 levels to Dpt extract and ConA-bound fraction were higher than to ConA-unbound fraction (p < 0.001) and the same was observed for positivity outcomes. When both groups were compared, IgG4 mean levels to Dpt extract and ConA-unbound fraction were higher in the group DP+ than in the group NA (p< 0.001), but no significant difference was found in the IgG4 levels to ConA-bound fraction between DP+ and NA groups (Figure 2(c)).

**Figure 3:**
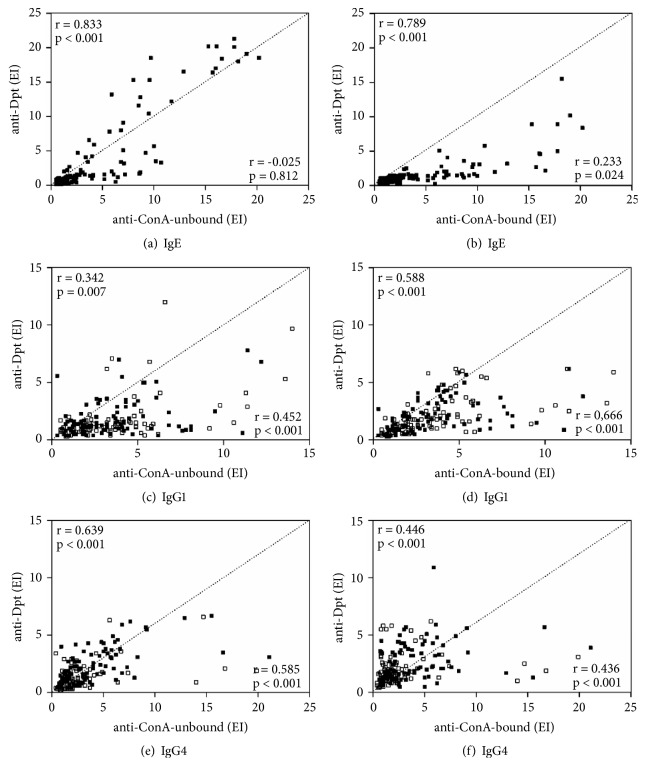
Correlation between levels of specific IgE (a, b), IgG1 (c, d), and IgG4 (e, f) to the* D. pteronyssinus* (Dpt) crude extract and ConA-unbound or ConA-bound fractions, expressed in ELISA index (EI) in sera from 95 patients with positive SPT to* D. pteronyssinus* (DP+ group; full squares, half left) and 92 nonallergic subjects (NA group; empty squares; half right). Correlation coefficient (r) was calculated using the Spearman correlation test.

Correlation analyses revealed high positive correlations between IgE levels to Dpt extract and ConA-unbound (r = 0.833; p < 0.001) or ConA-bound (r = 0.789; p < 0.001) fractions in the group DP+ (Figures 3(a)-3(b)). For IgG1 reactivity, moderate positive correlations were found between Dpt extract and ConA-bound fraction, in both DP+ (r = 0.588; p < 0.001) and NA (r = 0.666; p < 0.001) groups (Figure 3(d)). Similar positive correlations were observed between IgG4 levels to Dpt extract and ConA-unbound fraction in both groups (DP+: r = 0.342; p = 0.007; NA: r = 0.452; p < 0.001) (Figure 3(c)). Likewise, moderate positive correlations were observed between IgG4 levels to Dpt and ConA-bound fraction in the group DP+ (r = 0.446; p < 0.001) and group NA (r = 0.436; p < 0.001) (Figure 3(f)) as well as between Dpt and ConA-unbound fraction in the group DP+ (r = 0.639; p < 0.001) and group NA (r = 0.585; p < 0.001) (Figure 3(e)).

IgE/IgG1, IgE/IgG4, and IgG1/IgG4 ratios to Dpt extract and ConA-unbound and ConA-bound fractions were calculated in both DP+ and NA groups. In general, ConA-unbound and ConA-bound ratios were similar within these groups and only differences among Dpt extract and its fractions were seen.

**Figure 4:**
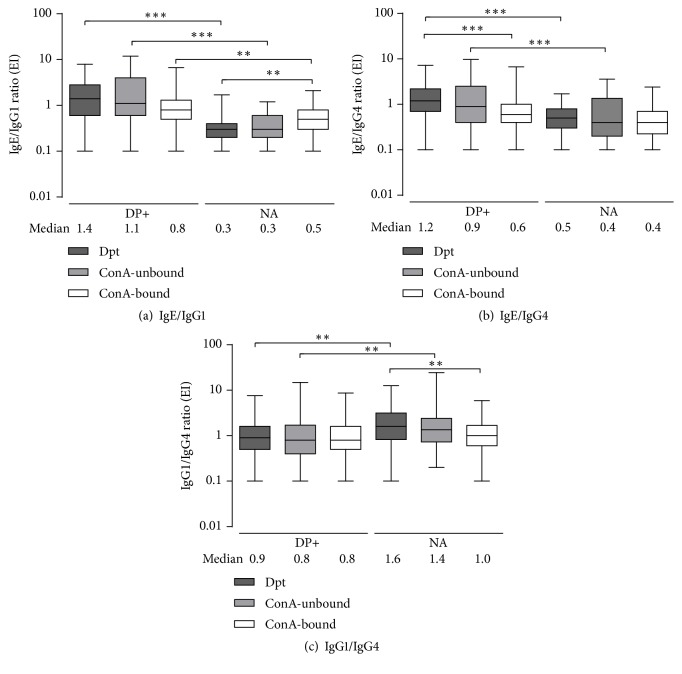
IgE/IgG1 (a), IgE/IgG4 (b), and IgG1/IgG4 (c) ratios to the* D. pteronyssinus* (Dpt) crude extract and ConA-unbound and ConA-bound fractions in sera from 95 patients with positive SPT to* D. pteronyssinus* (DP+ group) and 92 nonallergic (NA group) subjects. Boxes indicate the median with minimum and maximum values. Significant differences are reported in indicative bars. *∗∗*p < 0.01; *∗∗∗*p < 0.001.

In the group DP+, there was no difference between IgE/IgG1 ratios to ConA-unbound/ConA-bound fractions and the Dpt extract. However, IgE/IgG1 ratio to ConA-bound fraction was slightly higher than Dpt extract in the NA group (p < 0.01; Figure 4(a)). Group DP+ presented the higher IgE/IgG1 ratios compared to the group NA for all extracts (Figure 4(a)).

IgE/IgG4 ratio to Dpt extract was higher than ConA-bound fraction in the group DP+ (Figure 4(b)). When the distinct groups were compared, IgE/IgG4 ratio to both Dpt extract and ConA-unbound fraction were higher in the group DP+ than NA (Figure 4(b)).

A higher IgG1/IgG4 ratio to both Dpt and ConA-unbound fraction was found in the group NA when compared to DP+ group (Figure 4(c)). Moreover, ratios were also significantly lower to ConA-bound fraction than Dpt extract in the NA group (1.0 and 1.6, respectively; Figure 4(c)).

### 3.4. IgE, IgG1, and IgG4 Cross-Reactivity to Dpt Extract and ConA-Unbound and ConA-Bound Fractions

**Figure 5:**
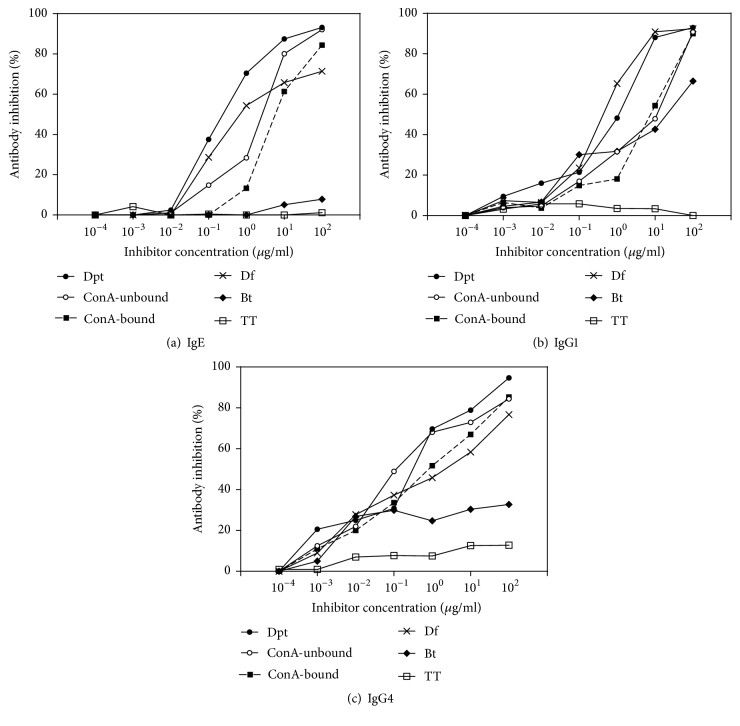
IgE (a), IgG1 (b), and IgG4 (c) inhibition curves using* D. pteronyssinus* (Dpt) crude extract on the solid phase, determined by ELISA. Pooled sera from DP+ patients were preabsorbed with different concentrations of Dpt, ConA-unbound, ConA-bound, Df crude extract, and Bt crude extract as inhibitors. Tetanus toxoid (TT) was used as unrelated antigen. Dashed line represents the half-maximal inhibitory concentration (IC_50_).

IgE, IgG1, and IgG4 cross-reactivity to Dpt extract and both ConA-unbound and ConA-bound fractions was assessed by ELISA using homologous (Dpt, ConA-unbound, and ConA-bound) and heterologous (crude Df and Bt extracts) inhibition. Tetanus toxoid (TT) was used as unrelated antigen. High homologous inhibition was observed to the Dpt extract and ConA-unbound and ConA-bound fractions for IgE (93%, 92%, and 84%, respectively, for Dpt, ConA-unbound, and ConA-bound; Figure 5(a)), IgG1 (93%, 91%, and 90%, respectively; Figure 5(b)), and IgG4 (95%, 84%, and 85%, respectively; Figure 5(c)) reactivities, at maximal concentration of 100 *μ*g/ml of each inhibitor. In addition, similar dose-response IgE inhibition curves were mostly observed between Dpt and Df extracts, with a half-maximal inhibition (I_50_) being reached at low concentrations of inhibitors (0.3 and 0.9 *μ*g/ml, respectively; Figure 5(a)). For IgG1, I_50_ values were closer in Dpt (0.7 *μ*g/ml) and Df (0.4 *μ*g/ml) (Figure 5(b)). For IgG4, closer I_50_ values were observed in Dpt extract (0.3 *μ*g/ml) and ConA-unbound fraction (0.1 *μ*g/ml) (Figure 5(c)). Thus, heterologous inhibition was higher with the Df extract (71% for IgE, 92% for IgG1, and 76% for IgG4 reactivity) than the Bt extract (8% for IgE, 66% for IgG1 and 32% for IgG4) at maximal concentration of inhibitors. Unrelated antigen (TT) exhibited negligible inhibition for IgE and IgG1 (1% and 0%, respectively) and low inhibition for IgG4 (12%).

### 3.5. IgE-, IgG1-, and IgG4-Binding to Polypeptides of Dpt Extract and ConA-Unbound and ConA-Bound Fractions

**Figure 6:**
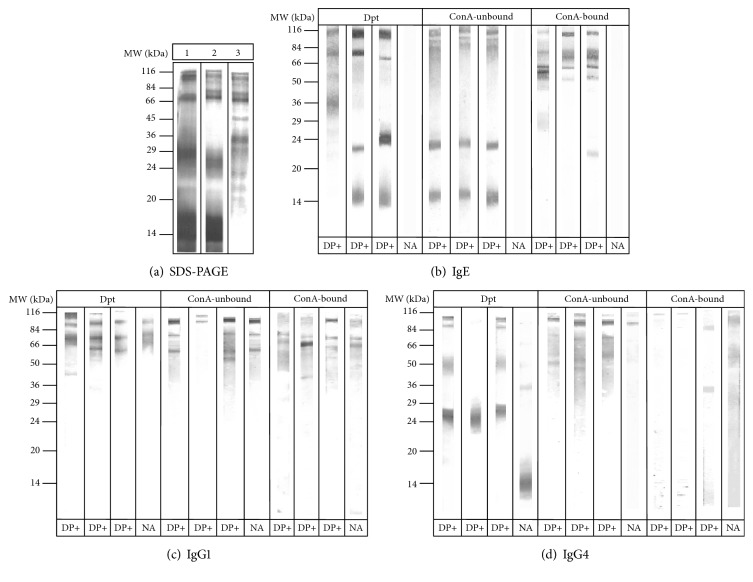
SDS-PAGE profile (a) of the* D. pteronyssinus* crude extract (Dpt, lane 1) and ConA-unbound (lane 2) and ConA-bound (lane 3) fractions on visualization by silver staining. Three representative sera from DP+ group and one from NA group used on Western Blotting for IgE (b), IgG1 (c), and IgG4 (d) are exposed. Protein standard molecular weights are indicated in kDa.

Polypeptide profiles of Dpt extract and ConA-unbound and ConA-bound fractions were visualized in 12% SDS-PAGE (Figure 6(a), lanes 1, 2, and 3, respectively). A broad range of bands were detected in the Dpt extract, although some of them were found thicker (14 kDa; 25 to 29 kDa) and others were found sharp, such as high molecular weight bands (66 kDa, 72 kDa, and 99 kDa). Some polypeptides from the Dpt extract were also found in the ConA-unbound fraction, particularly those above 66 kDa and below 20 kDa. Most of those high molecular weight bands are also found in the ConA-bound fraction, although a polypeptide profile complementary among both fractions has been noticed as prominent, particularly 32 kDa, 35 kDa, 45 kDa, 72 kDa, 80 kDa, 95 kDa, and 105 kDa.

**Figure 7:**
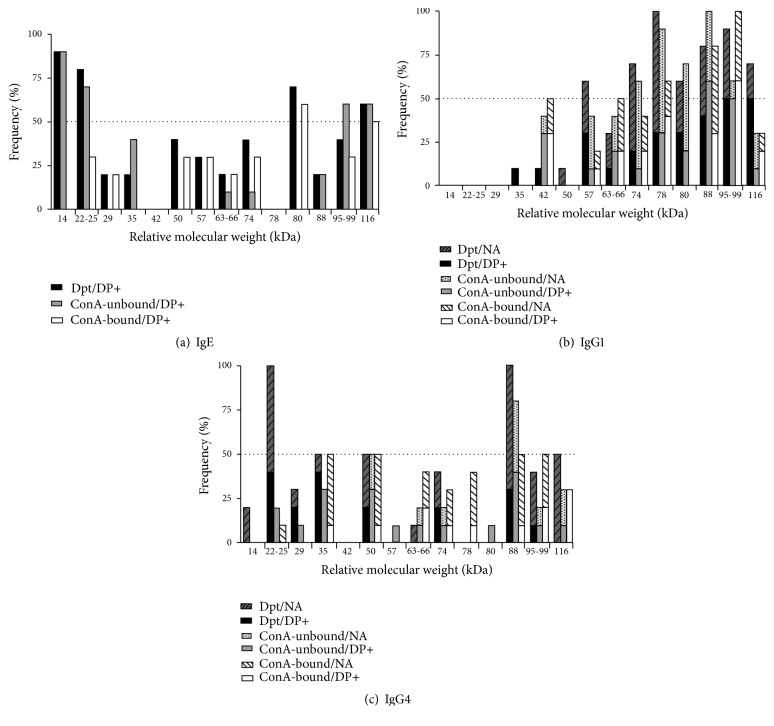
Frequency of polypeptide bands of* D. pteronyssinus* (Dpt) crude extract and ConA-unbound and ConA-bound fractions recognized by IgE (a), IgG1 (b), and IgG4 (c) antibodies in DP+ patients and nonallergic (NA) subjects. Relative molecular weights are indicated in kDa.

IgE-, IgG1-, and IgG4-binding activity of Dpt, ConA-unbound, and ConA-bound fractions was determined by Western Blotting in 10 serum samples from each DP+ and NA groups. Representative serum samples in each group and each extract for IgE (Figure 6(b)), IgG1 (Figure 6(c)), and IgG4 (Figure 6(d)) are demonstrated. Frequency of band recognition is also represented for IgE- (Figure 7(a)), IgG1- (Figure 7(b)), and IgG4- (Figure 7(c)) binding activity. Immunodominant bands (≥ 50%) were recognized by IgE, including the 14 kDa and 22-25 kDa bands in both Dpt and ConA-unbound fraction, 80 kDa in Dpt and ConA-bound fraction, 99 kDa in the ConA-unbound fraction, and 116 kDa in all extracts (Figure 7(a)). As expected, no polypeptide bands were recognized by IgE in the group NA. Interestingly, IgG1 mostly recognized bands above 42 kDa, with immunodominance for 57 kDa and 116 kDa bands in the Dpt, 80 kDa in the Dpt and ConA-unbound fraction, and 78 kDa, and 95-99 kDa for all extracts. Polypeptides with 35 and 50 kDa were weakly recognized by IgG1 (Figure 7(b)).

Band labeling by IgG4 was mostly faint in all extracts (Figure 6(d)), although frequently reaction was seen in sera from the group NA to the 22-25 kDa and 88 kDa bands in the Dpt extract (Figure 7(c)). Along with the 50 kDa band, the 88 kDa was predominantly recognized in all extracts. Only the 50 kDa and 88 kDa bands were immunodominant in the ConA-unbound fraction and same was seen in the ConA-bound fraction for 35 kDa, 50 kDa, 88 kDa, and 95-99 kDa bands. Besides, recognition of the 14, 29, 63-66, 74, 78, and 80 kDa bands was not very frequent.

## 4. Discussion

Glycoproteins often constitute immunogenic (innate immunity cells and T-cells) epitopes or IgE-binding sites in allergens [28, 29]. Instrumentally, ConA (from* Canavalia ensiformis*) lectin has been a valuable molecule for glycoconjugate purification as it commonly binds to D-mannose and D-glucose or its stereochemically related residues present in these molecules [30]. In our study, ConA-unbound and ConA-bound fractions were obtained from Dpt crude extract by ConA affinity chromatography and carbohydrate content found in both fractions revealed the presence of glycoconjugates even in the ConA-unbound fraction, with no affinity to ConA.

Up to date, at least 20 groups of* D. pteronyssinus *allergens have been characterized with different structural and biochemical properties, but IgE-binding capacity is the most prominent feature to distinguish among major, mid-term or minor allergens. Group 1 (Der p 1; Der f 1) and group 2 (Der p 2; Der f 2) major allergens are the most important sensitizing agents of* Dermatophagoides *species since these allergens are strongly recognized by IgE antibodies in allergic patients [8]. Although Der p 1 is a glycosylated molecule, it was mostly found in the ConA-unbound fraction (10.3% of the total protein content), suggesting that carbohydrate moieties present in the native form of this allergen might have poor affinity to ConA. Controversially, mannose was reported as a mediator on Der p 1 uptake by dendritic cells [31], but Al-Ghouleh and colleagues [32] found a poor reactivity to native Der p 1 using anti-mannose antibody whereas a stronger signal was seen using anti-1,3-Fucose. We did not perform qualitative assays in order to characterize this allergen in our samples, but it is possible that ConA is not appropriate for Der p 1 isolation, based on our quantitative findings. Despite that, group 3 and nonglycosylated allergens are described with same molecular weight as Der p 1 [33, 34]; thus, it is likely that the stack of protein observed within this range on SDS-PAGE and Western Blotting for both Dpt extract and ConA-unbound fraction has been composed by, at least, those three allergens. Furthermore, as expected, only background levels of Der p 2 were found in the ConA-bound fraction (0.8% of the total protein content), suggesting that this allergen might not be associated with ConA due to absence of glycosylation sites. In addition to that, another glycoprotein has been identified and characterized as a major allergen [35]. It is termed Der p 23, and it was referred as a 14 kDa-protein that is difficult to extract from the fecal pellets and, consequently, not easy to use in its native form for laboratory approaches. Additional studies, such as direct binding with specific antibodies and mass spectrometry are worthy of further considerations to provide a better characterization of these proteins.

The presence of carbohydrate moieties in HDM allergens has been strongly reported but the role of these molecules on IgE recognition has remained controversial. In our previous studies, fractionation of components from* Blomia tropicalis* crude extract using ConA affinity chromatography revealed that IgE response to the ConA-bound Bt fraction is similar to the crude Bt extract, indicating that this fraction is important for allergenicity [36]. On the other hand, a given structural approach has revealed a potential N-glycosylation site for the mature group 1 allergen, but this glycan has not affected Der p 1 and Der f 1 allergenicities on recombinant form since the position in which this sugar was found has not corresponded to a IgE-binding site [37].

Not only IgE but IgG antibodies are also important for allergy studies. Boost of IgG levels, particularly IgG4, during or after immunotherapy suggests a protective status against clinical symptoms, including a shift of the Th2 profile towards Th1, upregulation of immunosuppressive cytokines, and downregulation of IgE [12]. Moreover, we previously [38] revealed that* D. pteronyssinus*-specific IgG antibodies in allergic patients are able to inhibit reaction mediated by specific IgE. Based on these findings, we have evaluated IgE, IgG1, and IgG4 responses to Dpt extract and ConA-related fractions aiming to investigate if glycosylation could be important on IgE/IgG1 and IgE/IgG4 ratios, which are considered valuable parameters for immunotherapy. We revealed that allergic patients can exhibit similar IgG4 levels to both Dpt extract and glycosylated fraction, indicating a potential use of the ConA-bound fraction for immunosuppressive therapy. We also demonstrated that IgG1 antibodies are not relevant for the clinical status of allergy to* D. pteronyssinus* since no difference in the IgG1 levels has been found between allergic patients and healthy subjects. Furthermore, positive correlations found between IgE levels to the Dpt extract and ConA-related fractions among allergic patients reveal that both fractions can generate IgE response.

Due to differences in the molecular structure of its allergens, cross-reactivity between* Dermatophagoides* and* B. tropicalis* is considerably low [39]. However, the majority of allergic patients are cosensitized to* D. pteronyssinus* and* D. farinae*, mostly due to high cross-reactivity, 80% and 88%, observed in group 1 and group 2 allergens, respectively [40, 41]. Although we have not clearly identified those allergens in our samples, we have found a high IgE-binding inhibition between these* Dermatophagoides *species, by ELISA. In addition, there was a total cosensitization to* D. pteronyssinus* and* D. farinae, *determined by Skin Prick Test, as we previously noticed in our prevalence studies conducted in Brazil [42, 43], showing that Pyroglyphidae family could be considered an important risk factor for sensitization of our patients.

In the present study, IgE antibodies have recognized a reasonably high amount of polypeptide bands in Dpt extract and ConA-related fractions, indicating the presence of allergenic molecules in all of them, although ConA-bound fraction contains a reasonably small number of allergenic bands. Interestingly, we have shown that IgE antibodies recognize a broader range of polypeptide bands when compared to the other antibody classes, but IgG1 recognition has been noticed preferably by high molecular weight bands while only few polypeptide bands have had more than 50% of recognition frequency by IgG4. This might be a useful data for further studies involving IgE/IgG binary in the allergic response. Despite this, further identification of allergens, such as Der p 1, Der p 2 and Der p 23 in association to IgE and IgG levels, mainly in ConA-related fractions, should be important to reveal components that cause sensitization in these patients.

Taking together, our data indicate that fractionation of* D. pteronyssinus* crude extract into mannose/glucose-enriched and -depleted fractions implicates in distinct IgE and IgG recognition patterns, mainly revealing a predominance of IgG antibodies to mannose-enriched fraction, which indicates a protective potential of this fraction. Furthermore, as IgE is described to have poor affinity to carbohydrate moieties [44], it was not expected that ConA-bound fraction elicits higher IgE levels than ConA-unbound one. However, ability of ConA-bound fraction to elicit IgG levels is an important finding as it may constitute a source for immunotherapy by acting on upregulation of immunomodulatory components to reduce the allergic symptoms.

## 5. Conclusions

The isolation of native components from* D. pteronyssinus *using ConA affinity chromatography should be a potential tool for immunochemical analysis of allergenic and antigenic molecules. These studies may be useful to determine new perspectives for immunotherapy using natural allergens from HDM.

## Figures and Tables

**Table 1:** Total protein, carbohydrate, Der p 1, and Der p 2 contents in the *D. pteronyssinus *(Dpt) crude extract and ConA-unbound and ConA-bound fractions. Results are expressed in absolute values (*µ*g/ml) or allergen content in relation to the total protein content (%).

Sample	Protein^a^ (*µ*g/ml)	Carbohydrate^b^ (*µ*g/ml)	Protein/Carbohydrate ratio	Der p 1^c^ (*µ*g/ml/%)	Der p 2^c^ (*µ*g/ml/%)
Dpt	5,000	2,500	2.0	293/5.9	308/6.1
ConA-unbound	1,300	700	1.8	135/10.3	87/6.7
ConA-bound	500	200	2.5	25/5.0	4.2/0.8

^a^Determined by Lowry method. ^b^Determined by Dubois method. ^c^Determined by ELISA.

## Data Availability

All data that support our results are available upon request.
